# Supplementary material for: Chromothripsis during telomere crisis is independent of NHEJ, and consistent with a replicative origin
Source: Genome Res. 2019 May;29(5):737–49. doi: 10.1101/gr.240705.118 (PMC6499312; doi:10.1101/gr.240705.118)
Supplement: Supplemental Material [file supp_gr.240705.118_Supplemental_file_1.zip › contigs/annotated_contigs/DB51/contig.2.DB51_length_635_mean_cov_6.08818897638.docx]

**DB51_length_635_mean_cov_6.08818897638**

```
CGGGATCTGCAGCCCGCCATGCCTGAGCCTCCCACCCCCTCTGTGGGCTCCTGTGCAGCGGGAGCCTCCCCGATGAGCGCCGCCCCCTG
 >chr7:140656551-140656887 - E=2e-184
CTCCACGGCGCCCAGTCCCATCAATCACCCAAGGGCTGAGGAGTGCGGGCACACTGCACAGGACTGGCAGGCAGCTCCACCTGCAGCCC

CAGTGCAGGATCCACTAGGTAAAGCCAGCTGGGCTCCTGAGTCTGGTGGGGACGTGGAGAACCTTTATGTCTAGCTCAGGGATTGTAAA

TACACCAATCAGCACTCTGCATCTAGCTCAGGATTTGTGAATGCACCAATCCACACTCTGTATCT|AGCT|TATTAGACCAGTAGAGCG
                                                                  >chr7:141431766-14143
CAATAGTGTTCCTCTAGATCTCAAGAGTATCTGTTGGTACCTTAATGTCAGTGTTGACATTTAGAATACATCCATAGAGAAGTAACATA
2069 - E=1e-170
TTTTCTAAAGTATTTTAATTTAACTTCTTTAATGTTACCAGCCCAATGATGTTAATAAAATAATTGTGATAATTAGTTTCCAAGTTATA

TAATGATCTCTGTGCAAAAACAAAACTTACTGAGGTAAGAAATAAGCGTCTAACTGAAATGGAAGGGATGAAACGCTAAGGCCAGTGAA

GACGCAGTCCCTCA
```
